# The Economic and Epidemiological Impact of Focusing Voluntary Medical Male Circumcision for HIV Prevention on Specific Age Groups and Regions in Tanzania

**DOI:** 10.1371/journal.pone.0153363

**Published:** 2016-07-13

**Authors:** Katharine Kripke, Nicole Perales, Jackson Lija, Bennet Fimbo, Eric Mlanga, Hally Mahler, James McOllogi Juma, Emmanuel Baingana, Marya Plotkin, Deogratias Kakiziba, Iris Semini, Delivette Castor, Emmanuel Njeuhmeli

**Affiliations:** 1 Health Policy Project, Avenir Health, Glastonbury, Connecticut, United States of America; 2 Health Policy Project, Futures Group, Washington, District of Columbia, United States of America; 3 Tanzania National AIDS Control Program, Dar es Salaam, Tanzania; 4 Independent Consultant, Dar es Salaam, Tanzania; 5 U. S. Agency for International Development, Dar es Salaam, Tanzania; 6 Jhpiego, Dar es Salaam, Tanzania; 7 Joint United Nations Programme on HIV/AIDS, Dar es Salaam, Tanzania; 8 United States Department of Defense, Dar es Salaam, Tanzania; 9 Office of the U.S. Global AIDS Coordinator, Washington, District of Columbia, United States of America; 10 U. S. Agency for International Development, Washington, District of Columbia, United States of America; University of Pittsburgh, UNITED STATES

## Abstract

**Background:**

Since its launch in 2010, the Tanzania National Voluntary Medical Male Circumcision (VMMC) Program has focused efforts on males ages 10–34 in 11 priority regions. Implementers have noted that over 70% of VMMC clients are between the ages of 10 and 19, raising questions about whether additional efforts would be required to recruit men age 20 and above. This analysis uses mathematical modeling to examine the economic and epidemiological consequences of scaling up VMMC among specific age groups and priority regions in Tanzania.

**Methods and Findings:**

Analyses were conducted using the Decision Makers’ Program Planning Tool Version 2.0 (DMPPT 2.0), a compartmental model implemented in Microsoft Excel 2010. The model was populated with population, mortality, and HIV incidence and prevalence projections from external sources, including outputs from Spectrum/AIDS Impact Module (AIM). A separate DMPPT 2.0 model was created for each of the 11 priority regions. Tanzania can achieve the most immediate impact on HIV incidence by circumcising males ages 20–34. This strategy would also require the fewest VMMCs for each HIV infection averted. Circumcising men ages 10–24 will have the greatest impact on HIV incidence over a 15-year period. The most cost-effective approach (lowest cost per HIV infection averted) targets men ages 15–34. The model shows the VMMC program is cost saving in all 11 priority regions. VMMC program cost-effectiveness varies across regions due to differences in projected HIV incidence, with the most cost-effective programs in Njombe and Iringa.

**Conclusions:**

The DMPPT 2.0 results reinforce Tanzania’s current VMMC strategy, providing newfound confidence in investing in circumcising adolescents. Tanzanian policy makers and program implementers will continue to focus scale-up of VMMC on men ages 10–34 years, seeking to maximize program impact and cost-effectiveness while acknowledging trends in demand among the younger and older age groups.

## Introduction

Nearly 10 years after the first HIV cases were diagnosed in 1980, an inverse correlation was observed between HIV prevalence and male circumcision (MC) prevalence [1]. By 2007, randomized controlled trials in South Africa, Kenya, and Uganda had demonstrated that voluntary medical male circumcision (VMMC) conferred a 60% reduction in the risk of female-to-male HIV transmission [2–4]. The findings compelled the World Health Organization (WHO) and the Joint United Nations Programme on HIV and AIDS (UNAIDS) to support VMMC as a safe and effective method for HIV prevention. WHO and UNAIDS recommended the intervention be adopted and prioritized within a comprehensive HIV prevention package in 14 countries with high HIV prevalence and low MC prevalence [5]. Other preventive effects of MC are reduced risk of infection from ulcerative sexually transmitted infections [6–10].

According to the Tanzania HIV/AIDS and Malaria Indicator Survey 2011–12, HIV prevalence among males ages 15–49 is more than 50% higher among those who are uncircumcised (5.2%) than among those who are circumcised (3.3%). The prevalence of HIV among males in this age group also varies by urban (5.2%) versus rural (3.4%) populations, as well as by region: for example, Njombe (14.2%) is highly affected by HIV and Dodoma (3.7%) is less affected [11]. The prevalence of MC (71.4% nationally in 2012) varies by region (ranging from 27.5% to 99%), religion (ranging from 25% to 96.8%), and other socioeconomic factors. Men with secondary education or higher are more likely to be circumcised (87.5%) than men with no education (52%), as are men living in an urban location (94.2%) than those in a rural location (64.2%) and men in the highest wealth quintile (92.4%) than those in the lowest wealth quintile (64.8%) [11].

In 2008, a situational analysis was undertaken in six regions of Tanzania to inform the roll-out of a national VMMC program [12]. The results demonstrated high acceptance of MC across both traditionally circumcising and noncircumcising communities. Men and women primarily cited tradition, the reduced risk of acquiring sexually transmitted infections, and hygiene as reasons for accepting the practice. Among traditionally circumcising communities, MC is performed during childhood or adolescence and serves as an important rite of passage into adulthood. In noncircumcising communities, the preventive medical benefits are considered most important, and men are more likely to accept health facility-based circumcision than traditional circumcision [13, 14].

In 2009, a VMMC program was piloted in three regions: Iringa, Kagera, and Mbeya. Together, the findings from the situational analysis and lessons learned from the pilot program shaped the National Strategy for Scaling Up Male Circumcision 2010–2015. This strategy proposed scale-up of VMMC for males ages 10–34 in seven regions and one district in which there was low circumcision prevalence and high HIV prevalence: Iringa, Kagera, Mbeya, Mwanza, Rukwa, Shinyanga, Tabora, and Rorya district, in Mara region. Since 2010, several of these priority regions have been further subdivided to add the new regions of Geita, Katavi, Njombe, and Simiyu (11 total priority regions and Rorya district, in Mara) [15]. The lower age bound for VMMC scale-up in the strategy reflected the preferred age at circumcision (5 to 10 years) reported in the situational analysis, and the potential platform for strengthening adolescent and reproductive health services; the upper age bound was based on evidence from the situational analysis indicating that males over the age of 35 are relatively less receptive to VMMC. The country targets were later disaggregated into a primary target (males ages 10–24) and a secondary target (males ages 25–34) [12].

From July 2010 to October 2014, more than one million VMMCs were performed in Tanzania through the national program [16]. Through another modeling exercise, we estimated these VMMCs will avert 17,000 HIV infections in the general population by 2025, even if the VMMC program were not continued after 2014 [17]. To achieve this number of VMMCs, the program has actively worked to overcome barriers in service delivery and communication [18–20]. The majority of those barriers disproportionately affected men over age 25, such as the preference for discrete and private services, preference for male service providers, the requirement for abstinence in the postsurgical period, loss of income, and fear of pain associated with postsurgical erections. Across multiple settings, VMMC was also perceived as shameful for older men and/or married men with children [18, 21, 22].

As a result, more than 70% of VMMC clients reached through the national program since its inception in 2010 have been between the ages of 10 and 19 [19]. The relative uptake of VMMC among adolescents ages 10–19 is higher than the relative age distribution of uncircumcised males would indicate [23]. The converse is true for males ages 25–49. Similarly, progress toward national VMMC goals varied considerably across regions. This led policymakers to investigate the relative impact and cost-effectiveness of circumcising males within different age groups and in different geographic settings.

In 2013, the government expressed interest in understanding the impact of investing in VMMC through the national program, and the added benefit of doing so in a geographically-targeted way. It also looked to measure the impact of attracting fewer clients over age 25 than expected. This analysis examines the cost and epidemiological consequences of reaching specific age groups and subnational regions as part of Tanzania’s National VMMC Program.

## Methods

### DMPPT 2.0 Model

The Decision Makers’ Program Planning Tool (DMPPT) 2.0 model is described in detail in a separate manuscript in this collection [24]. Briefly, DMPPT 2.0 is a simple compartmental model, implemented in Microsoft Excel 2010, designed to analyze the effects of age at circumcision on program impact and cost. The DMPPT 2.0 model tracks the number of circumcised males in newborns and in each five-year age group over time, taking into account age progression and mortality. The model calculates discounted VMMC program costs and HIV infections averted in the population in each year in a user-specified VMMC scale-up strategy, compared with a baseline scenario in which the MC prevalence remains the same as it was. The baseline scenario assumes that traditional or other circumcisions that produced the baseline MC prevalence continue at the same rate as before the VMMC program was initiated.

### Tanzania data sources

A separate DMPPT 2.0 model was created for each of the 11 priority regions for VMMC scale-up (Geita, Iringa, Kagera, Katavi, Mbeya, Mwanza, Njombe, Rukwa, Shinyanga, Simiyu, and Tabora). The Mara region was not included, since VMMC scale-up within Mara is limited to a single district, and the required data were not available to populate the model at the district level. At the request of the country team, an additional model was created combining all 11 priority regions together but not including the rest of the country. National level estimates are not informative because of the high background prevalence of MC outside of the priority regions, but the country team wanted a single model that would collate the results for all of the regions where the VMMC program is being conducted.

All model inputs can be found in the supplemental materials (S1 and S2 Appendices). The DMPPT 2.0 model is populated with population, mortality, and HIV incidence and prevalence projections from an external source. To create the regional DMPPT 2.0 files, we first created regional Spectrum/AIM files based on the validated 2013 National Spectrum/AIM [25] file obtained from UNAIDS, downloaded on January 13, 2014. Spectrum/AIM is a model that projects population size, mortality, and HIV prevalence and incidence based on data empirically collected from the country. HIV prevalence data from sentinel surveillance sites used in Spectrum AIM to project HIV incidence and prevalence were categorized into the 11 regions plus the remainder of the country. HIV prevalence curves were fit to the surveillance data within each region using the R-trend fitting method in the EPP3 module within Spectrum/AIM [25]. Sites from several newly formed regions were grouped for the curve-fitting, because historical Demographic and Health Survey data only existed for the region prior to being split. Grouping the sites also meant that each curve-fitting was based on more data, making it more robust. The sites for Geita, Mwanza, Shinyanga, and Simiyu were grouped for the curve-fitting. The sites for Iringa and Njombe were also grouped, as were the sites for Katavi and Rukwa. Population by age and year, mortality by age and year, and annual number of male births were exported from the regional Spectrum/AIM files into their respective regional DMPPT 2.0 files.

For HIV incidence, we used a single national Spectrum/AIM file in which the surveillance sites had been categorized into regions, and calculated the incidence for each region or combination of regions as described above. HIV incidence by age from a national Spectrum/Goals model [11, 25] for Tanzania (S2 Fig) was multiplied by the ratio of the HIV incidence in each region (exported from the Spectrum/AIM file) to the national HIV incidence for each year between 2013 and 2020. For the years after 2021–2050, the regional to national incidence ratio for 2020 was used to scale the national incidence by age.

Numbers of VMMCs conducted in the country in each region in each year, disaggregated by age group, were extracted from the national health management information system on March 19, 2015. Male circumcision prevalence by age group in the model base year (2014) was based on the Tanzania HIV/AIDS and Malaria Indicator Survey 2011–12 [11]. The unit cost of VMMC used in the analysis was $83 USD (all subsequent references to currency are in 2014 U.S. dollars), based on [26] and validated by stakeholders in Tanzania. This is consistent with the 2014 costing study by Menon et al., which determined VMMC would cost $36–$128 depending on the region and service delivery model [27]. For ART we used a cost per patient year of treatment of $515 based on an international weighted average median price in 2011 of $145 for first- and second-line antiretroviral drugs [28]; $222 for average service delivery and monitoring costs [29]; plus an additional 40% for costs above the facility level for administration, logistics, training, planning, and so forth.

### Analytical approaches

To examine the effect of client age on the impact of scaling up VMMC, we created a series of scenarios in which each scenario had a target of 80% MC prevalence for a single age group or combination of age groups, leaving the target for the other age groups at the same level as the baseline. We created one scenario for each individual five-year age group, in addition to several scenarios with 80% targets for combined age groups, such as 10- to 34-year-olds or 15- to 29-year-olds. In each scenario, MC coverage was scaled up between 2014 and 2018 by applying a linear interpolation to the baseline MC prevalence for each age group in 2013 and the target coverage in 2018. After 2018, the coverage for each age group was maintained at 80%. For each scenario, we compiled the decrease in HIV incidence in the scale-up scenario compared with the HIV incidence in the baseline scenario in each year of the model, and the total number of VMMCs required during the scale-up phase (2014–2018). The following model outputs for each scenario were measured over the 15-year period between 2014 and 2028, inclusive: the total number of HIV infections averted in the population (including secondary infections averted among females; see above), the number of VMMCs per HIV infection averted, and the total cost of the VMMC program. Costs, numbers of VMMCs (when calculating VMMC per HIV infection averted), and infections averted were all discounted at a rate of 3% per year. Uncertainty around the age distribution of HIV incidence was calculated as described in a separate manuscript in a separate manuscript in this collection [24], except that the incidence for each age group was varied by +/-15%, based on the estimated variance in incidence by age from the ALPHA Network [30] incidence rate ratios by age used in Spectrum/AIM.

To examine the differences in impact and cost-effectiveness across the different regions of Tanzania, we compiled the cost per HIV infection averted over the 15-year period 2014–2028, inclusive, from each regional DMMPT 2.0 file. The VMMC scale-up scenario used for this analysis was to scale up to 80% coverage among males ages 10–34. Uncertainty around the regional estimates was calculated as follows: The Spectrum/AIM Uncertainty Analysis tool was run on each individual regional Spectrum/AIM file described above. The median, lower 2.5%, and upper 97.5% bounds around the adult (ages 15–49) HIV incidence for the year 2020 were extracted from the Uncertainty tool. DMPPT 2.0 files for each region were created representing the lower and upper bounds of the HIV incidence by multiplying the incidence in the original file by the ratio of the lower to median or upper to median values. Cost per HIV infection averted extracted from the lower bound file for each region was used as the lower bound in the analysis, and likewise for the higher bound.

## Results

We considered four metrics related to circumcision of different client age groups: immediacy of impact, magnitude of impact, cost-effectiveness, and number of VMMCs per HIV infection averted. In this analysis, immediacy of impact is measured by the relative reduction in HIV incidence over a 5-year period, magnitude of impact is measured by the relative reduction in HIV incidence over a 15-year period, cost-effectiveness is measured by the discounted cost per HIV infection averted over a 15-year period, and the number of VMMCs per HIV infection averted is measured in a 15-year period. All metrics are measured starting in the year 2014.

**Fig 1:**
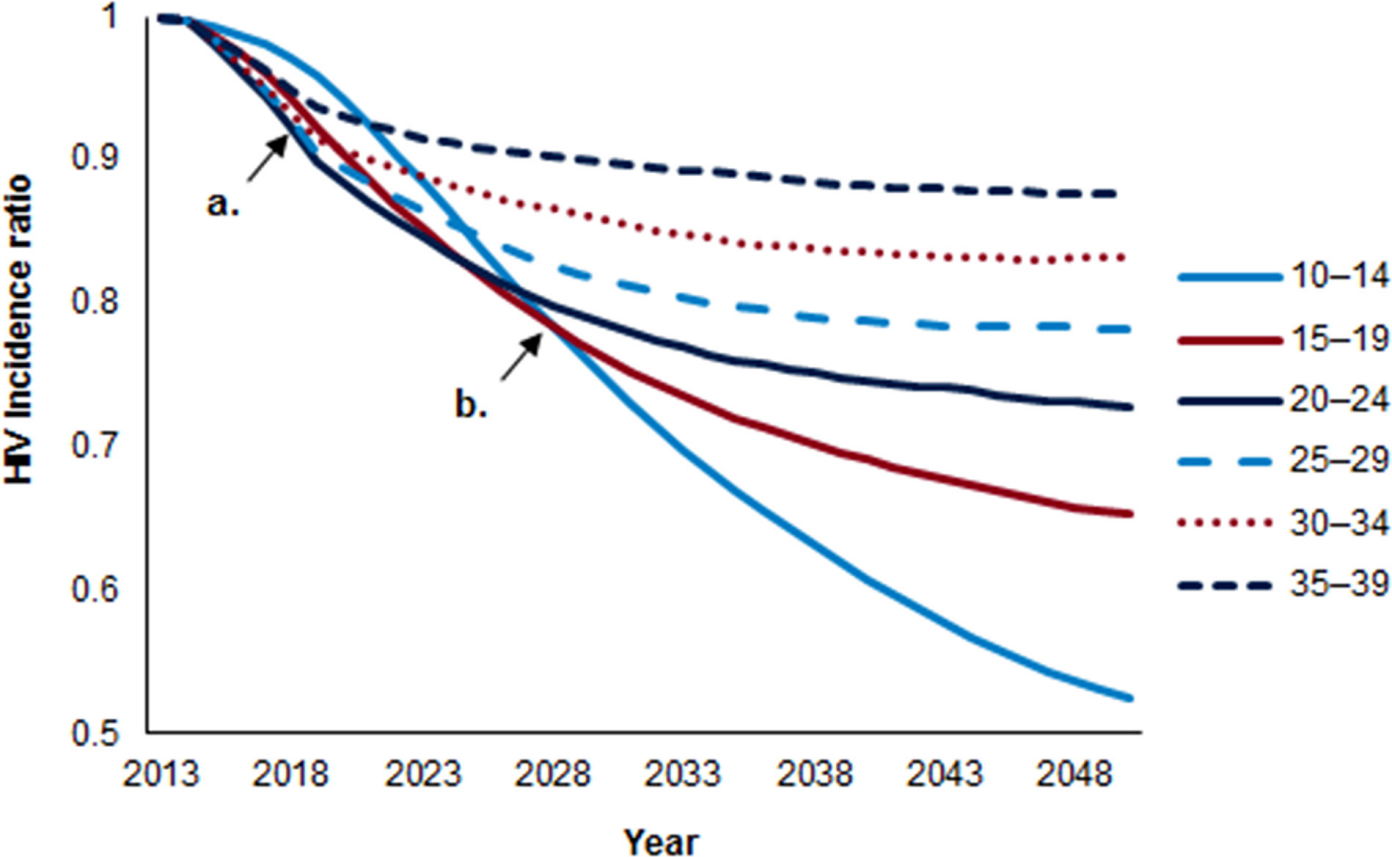
Modeled relative reduction in HIV incidence by scaling up VMMC for individual age groups, compared with no scale-up of VMMC over baseline levels, 2014–2050. **(a) immediacy of impact (5 years). (b) magnitude of impact (15 years).** The HIV incidence ratio represents the incidence in the scale-up scenario divided by the HIV incidence in a population where circumcision is not scaled-up over baseline levels. Each line represents the HIV incidence ratio under a scenario in which only the indicated five-year age group is circumcised. Marker a represents a five-year period from the base year (2014). Marker b represents a 15-year period from the base year.

To look at immediacy of impact, we looked at the relative reduction in HIV incidence achieved after five years, by circumcising individual five-year age groups of clients compared with a baseline scenario that does not provide VMMCs (Fig 1). The figure presents the reductions in HIV incidence in the general population over 2014–2050 that would be achieved by scenarios in which only clients in an indicated five-year age group are circumcised. The immediacy of impact (five years) is largely a function of the projected HIV incidence in the male subpopulation targeted. The highest HIV incidence among Tanzanian males is projected to occur among those in the 20- to 34-year-olds [24]; the most immediate reduction in HIV incidence is projected to result from VMMC scale-up among males in the 20- to 24-, 25- to 29-, and 30- to 34-year-olds.

Although in the short term (five years), circumcising males in the 20- to 24-, 25- to 29-, and 30- to 34-year-olds is projected to produce the greatest reduction in HIV incidence, over a 15-year period, circumcising younger age groups—those 10- to 14-, 15- to19-, and 20- to 24- years old—is projected to have the greatest impact on HIV incidence (Fig 1). This is because males who are circumcised before sexual debut are protected for the longest proportion of their lifetime HIV exposure, and because the younger age groups are larger in population size.

**Fig 2:**
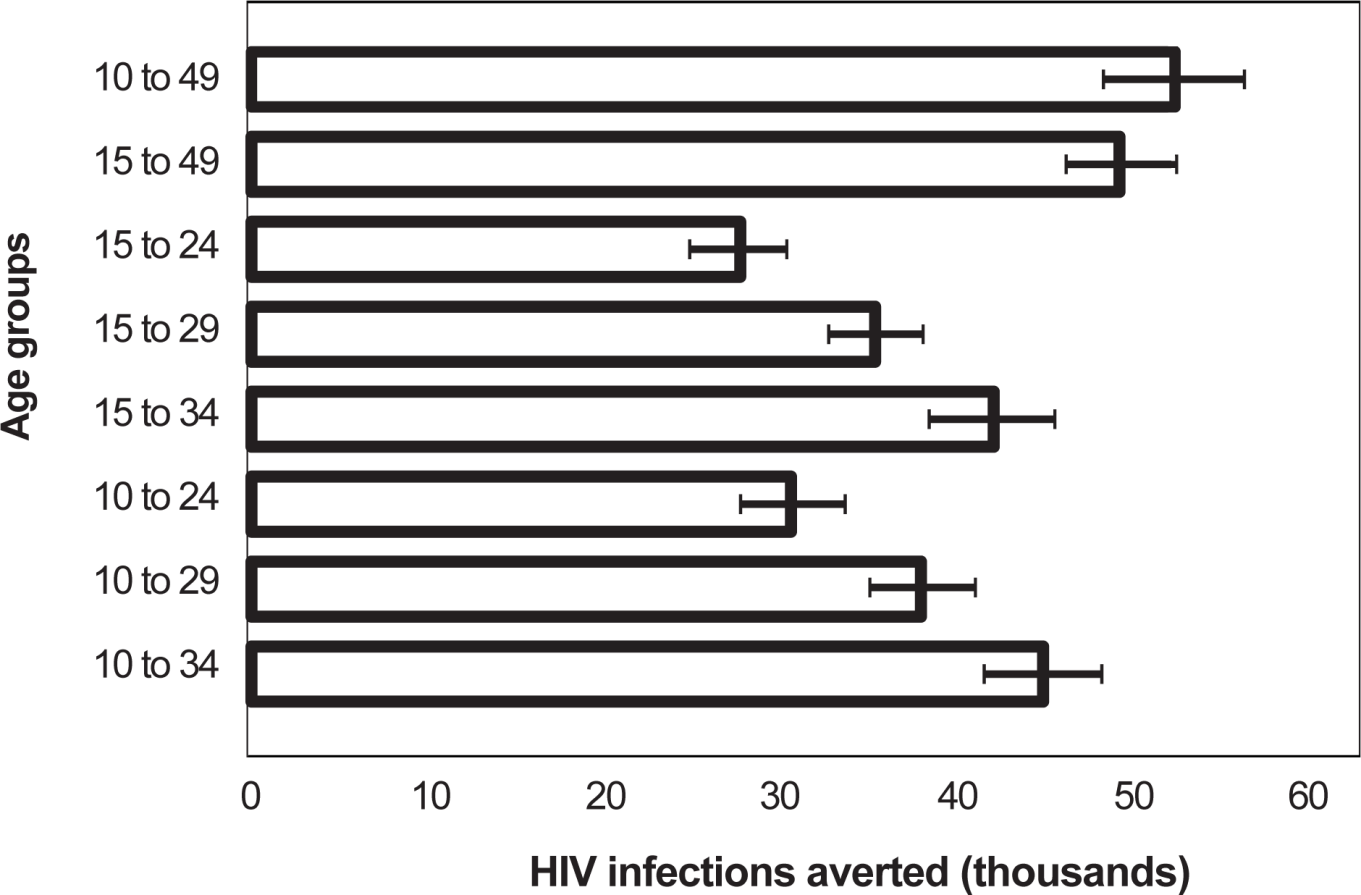
HIV infections averted in scenarios scaling up VMMC among different client age groups. The time period for measuring HIV infections averted was 15 years, 2014–2028, inclusive. Error bars represent uncertainty bounds.

The previous analyses examined scenarios in which only a single five-year age group at a time is circumcised. To assess the impact and cost-effectiveness of circumcising age groups that might be closer to actual implementation strategies, we examined several scenarios with 80% targets for combined age groups, such as 10- to 34-year-olds or 15- to 29-year-olds. Fig 2 considers the impact of targeting combined age groups for VMMC scale-up. The DMPPT 2.0 model tracks circumcised males as they transition into older age groups over time. Hence, the results in Fig 2 are not the aggregate of results derived from circumcising individual five-year age groups. This analysis shows that the greatest number of HIV infections averted is achieved with the broadest age group: males 10–49 years old. This is the age group with the largest number of clients, so it results in the greatest number of HIV infections averted. Circumcising males ages 15–49 results in slightly fewer HIV infections averted. If programmatic challenges make reaching males ages 35–49 impossible, 88% of HIV infections could still be averted by circumcising males ages 10–34.

**Fig 3:**
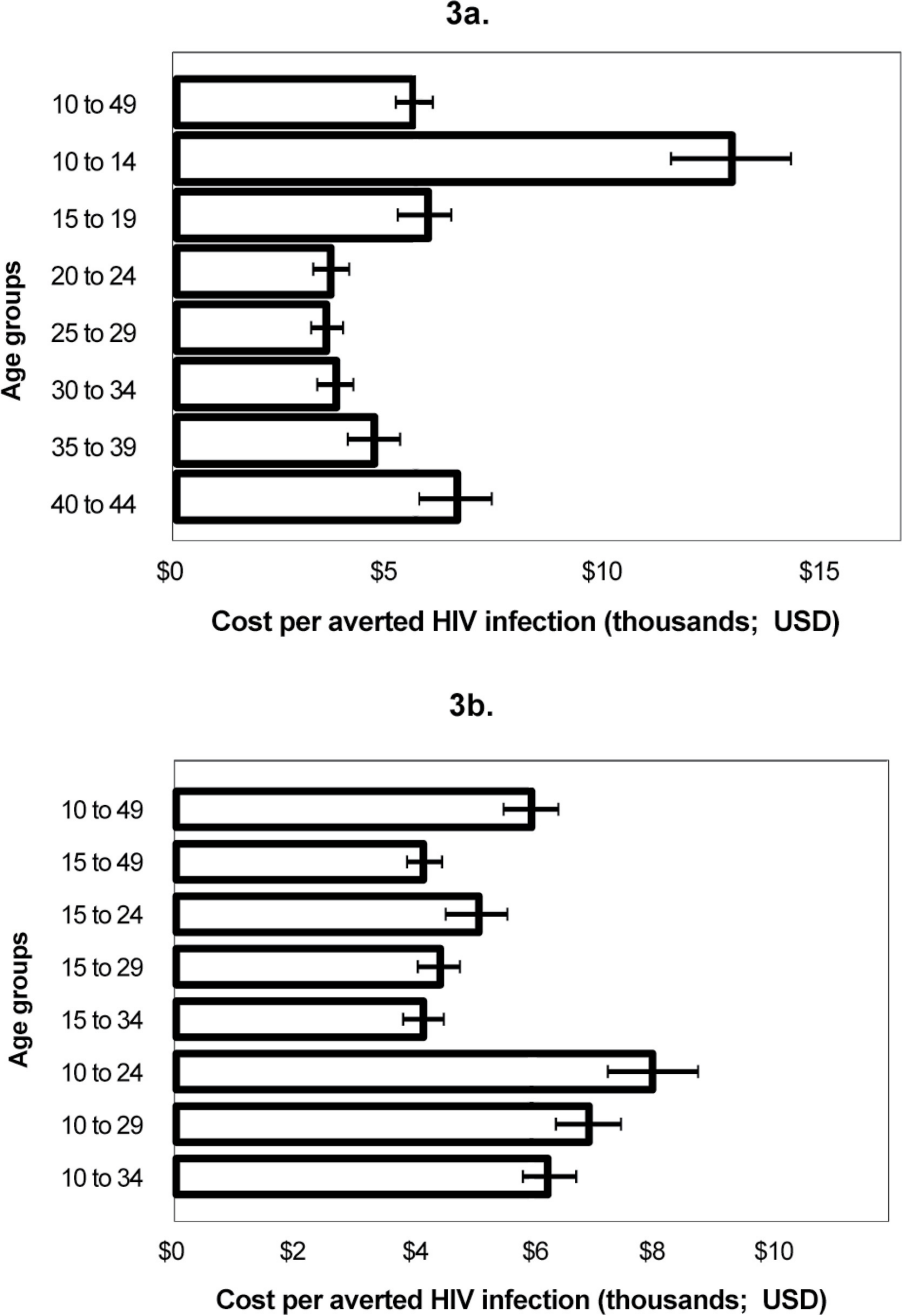
**a and b. Cost per HIV infection averted in scenarios scaling up VMMC among different client age groups.** The time period for measuring HIV infections averted was 15 years, 2014–2028, inclusive. Error bars represent uncertainty bounds.

Fig 3A and 3B compare the cost-effectiveness of circumcising five-year and broader age groups of clients. Circumcising males ages 20–24, 20–29, or 30–34 would achieve the lowest cost per infection averted, compared to circumcising males ages 10–49 (Fig 3A). In the combined age groups depicted in Fig 3B, the lowest cost per HIV infection averted would be achieved when circumcising males ages 15–34, but uncertainty bounds indicate that circumcising males ages 15–29 or 15–49 could be equally or more cost-effective. Although strategies that include males ages 10–14 lead to higher costs per HIV infection averted (less cost-effectiveness), historical program data indicate that males ages 10–14 constitute a large proportion of VMMC clients irrespective of targeted demand creation. By circumcising males ages 10–29 or 10–34, the program would achieve the same level of cost-effectiveness as circumcising males ages 10–49, given the uncertainty bounds. This analysis does not consider variations in cost across age groups due to differences in demand creation or service delivery.

Table 1 summarizes the priority age group for each metric examined. VMMC scale-up to 80% MC coverage for males ages 20–24, 25–29, and 30–34 by 2018 would result in the fewest VMMC per HIV infection averted [24] and the most immediate impact on HIV incidence. This scenario would require 0.72 million VMMCs over 2014–2018. Over a 15-year period, the age groups that produce the greatest decrease in HIV incidence are 10- to 14-, 15- to 19-, and 20- to 24-year-olds, so these age groups are prioritized for magnitude of impact. The most cost-effective program, providing the lowest cost per HIV infection averted, would circumcise 80% of males ages 15–34 and would require 1.07 million VMMCs by 2018.

**Table 1:** Priority age groups and number of VMMCs required for each parameter in the model framework, Tanzania.

Parameter	Priority age group	No. VMMCs required to reach 80% MC coverage by 2018
VMMC/HIV Infection Averted	20–34	0.72 million
Immediacy of Impact	20–34	0.72 million
Magnitude of Impact	10–24	1.69 million
Cost-effectiveness	15–34	1.07 million
Country Age Targeting Strategy	10–34	2.16 million

In comparison, circumcising 80% of males ages 10–49 would require 2.57 million VMMCs over 2014–2018 and the current age strategy in Tanzania would require 2.16 million VMMCs over 2014–2018.

**Fig 4:**
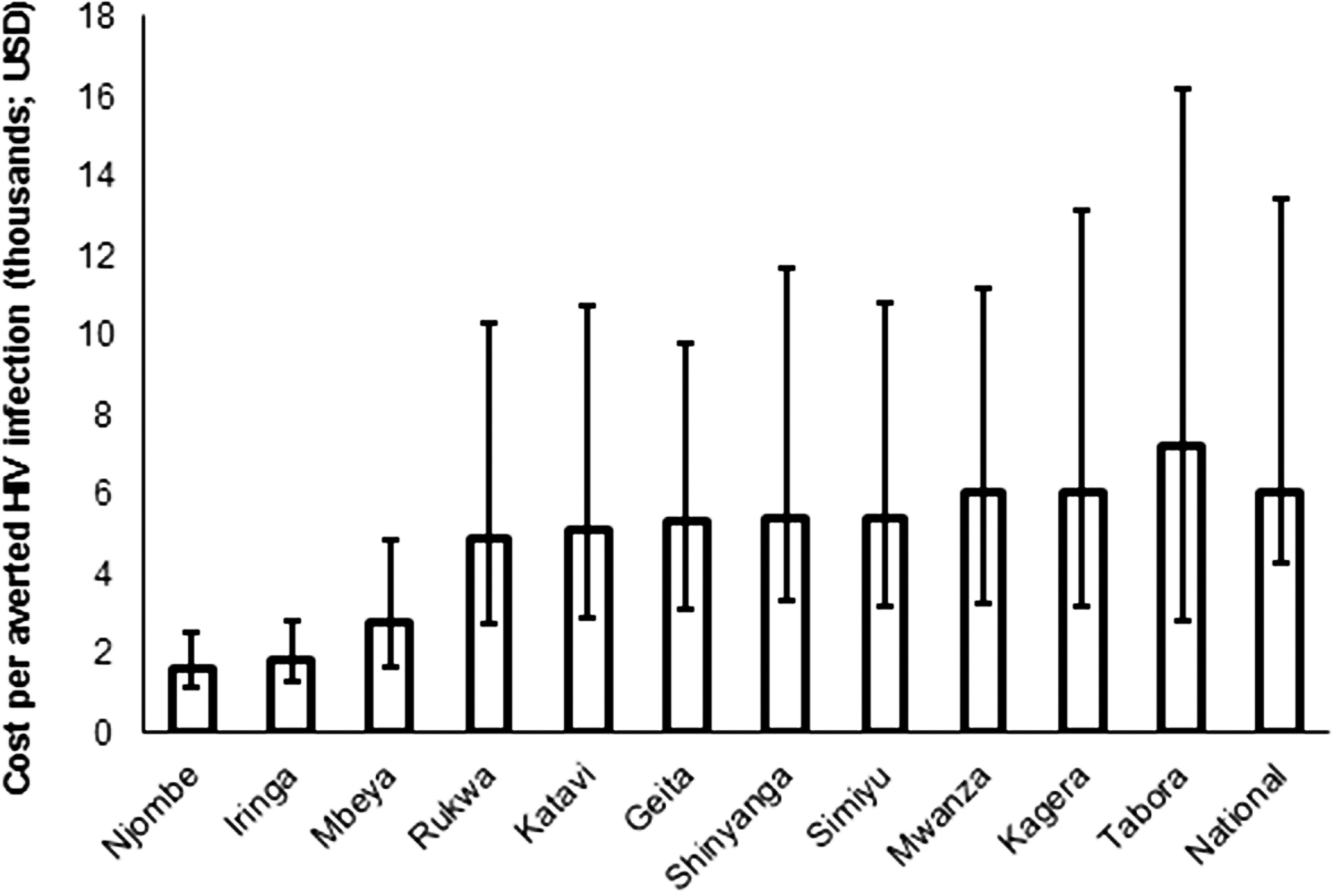
Discounted cost per HIV infection averted by region, 2014–2028, given a scenario of scale-up to 80% of 10- to 34-year-olds.

The Tanzania National VMMC Program is already prioritized geographically, by focusing on the 11 priority regions plus Rorya district, in Mara. We assessed whether further prioritization of the 11 priority regions would be advisable, by examining how the cost per HIV infection averted varied among the regions. Fig 4 compares the cost-effectiveness of scaling up VMMC within each of 11 priority regions. Cost-effectiveness varies across regions, due to regional differences in the projected HIV incidence. The model predicts that the most cost-effective VMMC programs will be in Njombe and Iringa, where the projected HIV incidence ranges from 0.6% to 1.1%. In the nine remaining regions, the projected HIV incidence ranges from 0.1% to 0.3% and cost-effectiveness of the VMMC programs therein is not distinguishable from the national estimate, considering uncertainty bounds. Despite the range in cost-effectiveness, the discounted HIV treatment costs averted by scaling up VMMC outweigh the projected discounted VMMC program costs in every region (S2 Fig). In other words, the VMMC program is cost saving in all 11 priority regions compared with HIV treatment costs averted.

**Fig 5:**
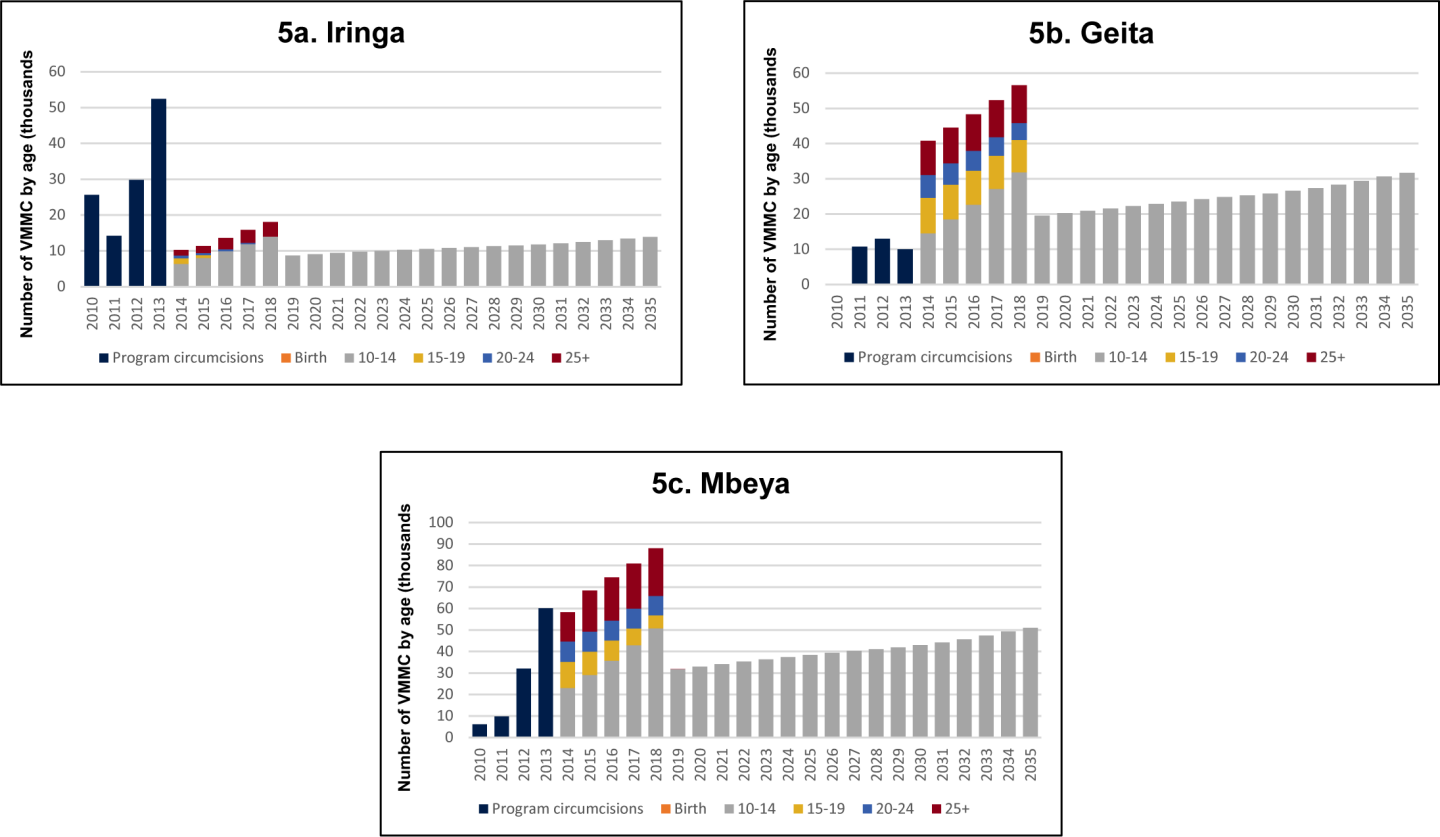
a–c. Annual VMMCs required to scale up to 80% MC coverage among males ages 10–34.

In Tanzania, the national program has been successful in scaling up VMMC through 2014, but regions differ in their progress toward the 80% VMMC coverage target in 2018. In Fig 5A–5C, the bars 2013 and prior represent actual VMMCs conducted by the program. From 2014, the model projects the number of VMMCs needed, by age group, to scale up to 80% coverage among males ages 10–34 by 2018 and maintain 80% coverage thereafter. The trends suggest Mbeya can easily reach the 2018 targets if it continues to scale up VMMC. Geita would likely have difficulty reaching 80% coverage among males ages 10–34 by 2018 unless it significantly expands services. Iringa has nearly reached the target and is already transitioning into the maintenance phase.

## Limitations

DMPPT 2.0 relies on available national and regional estimates of demographic and epidemiological data, and therefore modeled projections are subject to the assumptions and measurement errors of these inputs: baseline MC prevalence, unit costs, and projections of future HIV incidence. Future HIV incidence is especially uncertain, because it is dependent on many factors that are impossible to predict.

Cost assumptions were based on a fixed unit cost of $83 and therefore did not reflect differences in cost depending on client age, geographic location, implementation model, phase of scale-up, or other factors. In Tanzania, VMMC unit cost may be higher for men ages 20–34 and even higher for men ages 35 and older. Programmatic experience to date confirms these men would require more demand creation activities and health facility accommodations to increase privacy and adapt to lower volume settings. Higher unit costs would negatively affect the cost-effectiveness of the respective age groups. Total cost estimates for scale-up scenarios are therefore useful only for relative comparisons, and should not be used for budget projections.

Other factors transcending age and geography affect the implementation of the national VMMC program. In Tanzania, stigma associated with VMMC for married men with children, regardless of age, has been documented [21, 22]. The model does not account for broader social, cultural, and logistical barriers to program acceptability and implementation. When selecting a scale-up strategy or target, Tanzania will need to consider the model results in the context of these and other influences, such as human resources, the political environment, and challenges to demand creation specific to certain geographic areas or subpopulations.

## Discussion

The results of this analysis demonstrate that Tanzania can maximize HIV incidence reduction and cost-effectiveness over a 15-year period, by circumcising males ages 10–24 and 15–34, respectively. The DMPPT 2.0 modeling results also confirm that the VMMC program will be cost saving in the 11 priority regions, with programs in Njombe and Iringa regions being especially cost-effective. Given evidence that circumcision status may be associated with lower risk for certain sexually transmitted diseases in men and women, the HIV cost saving estimates reported here would underestimate the secondary benefits from reduced levels of sexually transmitted diseases [6–10, 31].

Tanzania was unique in its response to the global call by WHO and UNAIDS in 2007 to scale up VMMC as an effective intervention for HIV prevention. At the time, global stakeholders had not recommended age-specific targeting for national programs. Rather, in 2011 UNAIDS released the *Joint Strategic Action Framework to Accelerate the Scale-Up of VMMC for HIV Prevention in Eastern and Southern Africa*, which aimed for priority VMMC countries to achieve 80% circumcision prevalence among males ages 15–49 by 2016 [32]. Contrary to such guidance, the Tanzania National VMMC Program launched in 2010 focused on males ages 10–34 in the VMMC priority regions. In the absence of modeling for subpopulation targeting scenarios, the Ministry of Health and Social Welfare relied on the thorough situational analysis and lessons learned from a pilot program to determine the target age group. The ministry based its decision on cultural preferences, the potential of VMMC programs to serve as a platform for strengthening adolescent and reproductive health services, and the high cost and minimal success of attracting older clients.

The DMPPT 2.0 results reinforced the target set forth in the National Strategy for Scaling-Up Male Circumcision 2010–2015. As a result of this analysis, Tanzanian policy makers and program implementers chose to continue to focus scale-up of VMMC on males ages 10–34 years, thereby maximizing impact and cost-effectiveness while acknowledging the programmatic realities related to demand among the younger and older age groups. Although the age target went unchanged, the new evidence base would profoundly impact VMMC program implementation going forward. Tanzania’s early deviation from international recommendations generated some hesitancy among implementing partners to focus efforts and resources on younger age groups. The findings from this analysis empowered the national program and implementing partners to adopt a focused approach to service delivery tailored to males in the 10- to 34-year-olds, and especially adolescents.

The Tanzania National VMMC Program is now looking toward the implementation of its second circumcision strategy, the *VMMC Country Operational Plan 2014–2017*. The Country Operational Plan maintains its predecessor’s goal to achieve 80% circumcision prevalence among males ages 10–34. The refocus on adolescent VMMC services is reflected in the plan, which explicitly calls for heavy investments in “properly serving adolescents and their gatekeepers” [12]. The findings of this analysis also emphasize the importance of circumcision in younger populations to the long-term impact of VMMC on HIV incidence. As a result, the Ministry of Health and Social Welfare also defines three implementation phases of the Country Operational Plan: scale-up, catch-up, and sustainability. The scale-up and catch-up phases refer to the expansion of fixed sites and outreach schedules, while the sustainability phase entails a gradual transition toward VMMC for boys turning age 10 and early infant MC. By 2017, the country aims for all 12 regions to enter the sustainability phase [12].

The use of these results in Tanzania to inform ongoing implementation and the VMMC Country Operational Plan 2014–2017 exemplifies the Tanzania VMMC program’s commitment to applying evidence to improve policy, implementation, and the well-being of the Tanzanian population.

## Supporting Information

S1 Appendix Tanzania Model Inputs. (DOCX)

S2 Appendix Spectrum Inputs. (XLSX)

S1 Fig Age-specific male HIV incidence, 2013, from Spectrum/Goals model in Tanzania’s 11 priority regions. (TIF)

S2 Fig Comparison of VMMC program costs and HIV treatment costs averted, by region 2014–2028. (TIF)

## Abbreviations

DMPPT: Decision Makers’ Program Planning Tool
MC: male circumcision
UNAIDS: Joint United Nations Programme on HIV/AIDS
USD: U.S. dollars
VMMC: voluntary medical male circumcision
WHO: World Health Organization
