# Supplementary material for: The Economic and Epidemiological Impact of Focusing Voluntary Medical Male Circumcision for HIV Prevention on Specific Age Groups and Regions in Tanzania
Source: PLoS One. 2016 Jul 13;11(7):e0153363. doi: 10.1371/journal.pone.0153363 (PMC4943708; doi:10.1371/journal.pone.0153363)
Supplement: S1 Appendix — (DOCX) [file pone.0153363.s001.docx]

| Input | Data | Reference |
| --- | --- | --- |
| Percent circumcised in base year, by 5-year age group | EIMC: 5.0%  5-9: 5.0%  10-14: 21.7%  15-19: 40.4%  20-24: 48.5%  25-29: 40.5%  30-34: 45.6%  35-39: 47.1%  40-44: 43.4%  45-49: 36.9%  50-54: 36.9%  55-59: 36.9% | Tanzania HIV/AIDS and Malaria Indicator Survey 2011-12 (age >15 years)  Authors’ assumptions (EIMC, 5-9, 10-14) |
| Percent of historical program VMMC, by 5-year age group | EIMC: 0.0%  5-9: 0.8%  10-14: 44.6%  15-19: 33.0%  20-24: 11.8%  25-29: 4.2%  30-34: 2.5%  35-39: 1.5%  40-44: 0.7%  45-49: 0.4%  50-54: 0.3%  55-59: 0.1% | National Health Management Information System |
| Number of male circumcisions Performed, 2009-2013 | 2009: 871  2010: 36,814  2011: 118,743  2012: 183,616  2013: 266,091 | National Health Management Information System |
| VMMC effectiveness | 0.6 | Auvert B, Taljaard D, Lagarde E, Sobngwi-Tambekou J, Sitta R, et al. (2005) Randomized, controlled intervention trial of male circumcision for reduction of HIV infection risk: The ANRS 1265 trial. PLoS Med 2(11): e298.  Gray RH, Kigozi G, Serwadda D, Makumbi F, Watya S, Nalugoda N et al. Male circumcision for HIV prevention n men in Rakai, Uganda: a randomized trial. Lancet 2007; 369: 767-66.  Bailey RC, Moses S, Parker CB, Agot K, Maclean I, Krieger JN, et al. Male circumcision for HIV prevention in young men in Kisumu, Kenya: a randomized controlled trial. Lancet 2007; 369: 643-56. |
| Ratio of infections averted among females to males | 0.63 | Spectrum, Goals Model |
| Cost of ART per year | $515 | WHO, UNAIDS, UNICEF. Global HIV/AIDS response: epidemic update and health sector progress towards universal access: progress report 2011. World Health Organization, 2011.  Menzies NA, Berruti AA, Blandford JM. The determinants of HIV treatment costs in resource limited settings. PLoS One. 2012; 7(11):e48726.  Authors’ assumptions and calculations |
| Cost per VMMC procedure, by 5-year age group | EIMC: $41.28  5-9: $82.56  10-14: $82.56  15-19: $82.56  20-24: $82.56  25-29: $82.56  30-34: $82.56  35-39: $82.56  40-44: $82.56  45-49: $82.56  50-54: $82.56  55-59: $82.56 | Njeuhmeli E, Forsythe S, Reed J, Opuni M, Bollinger L, Heard N, et al. Voluntary medical male circumcision: modeling the impact and cost of expanding male circumcision for HIV prevention in eastern and southern Africa. PLoS Med. 2011;8(11):e1001132.  Stakeholder consultation. |
| HIV incidence | See S2 Appendix – Spectrum Inputs | Authors’ Calculations  Spectrum, AIDS Impact Module and Goals module |
| Discount rate | 0.03 | Authors’ assumption. |
