# Supplementary figures and images for: The Economic and Epidemiological Impact of Focusing Voluntary Medical Male Circumcision for HIV Prevention on Specific Age Groups and Regions in Tanzania
Source: PLoS One. 2016 Jul 13;11(7):e0153363. doi: 10.1371/journal.pone.0153363 (PMC4943708; doi:10.1371/journal.pone.0153363)

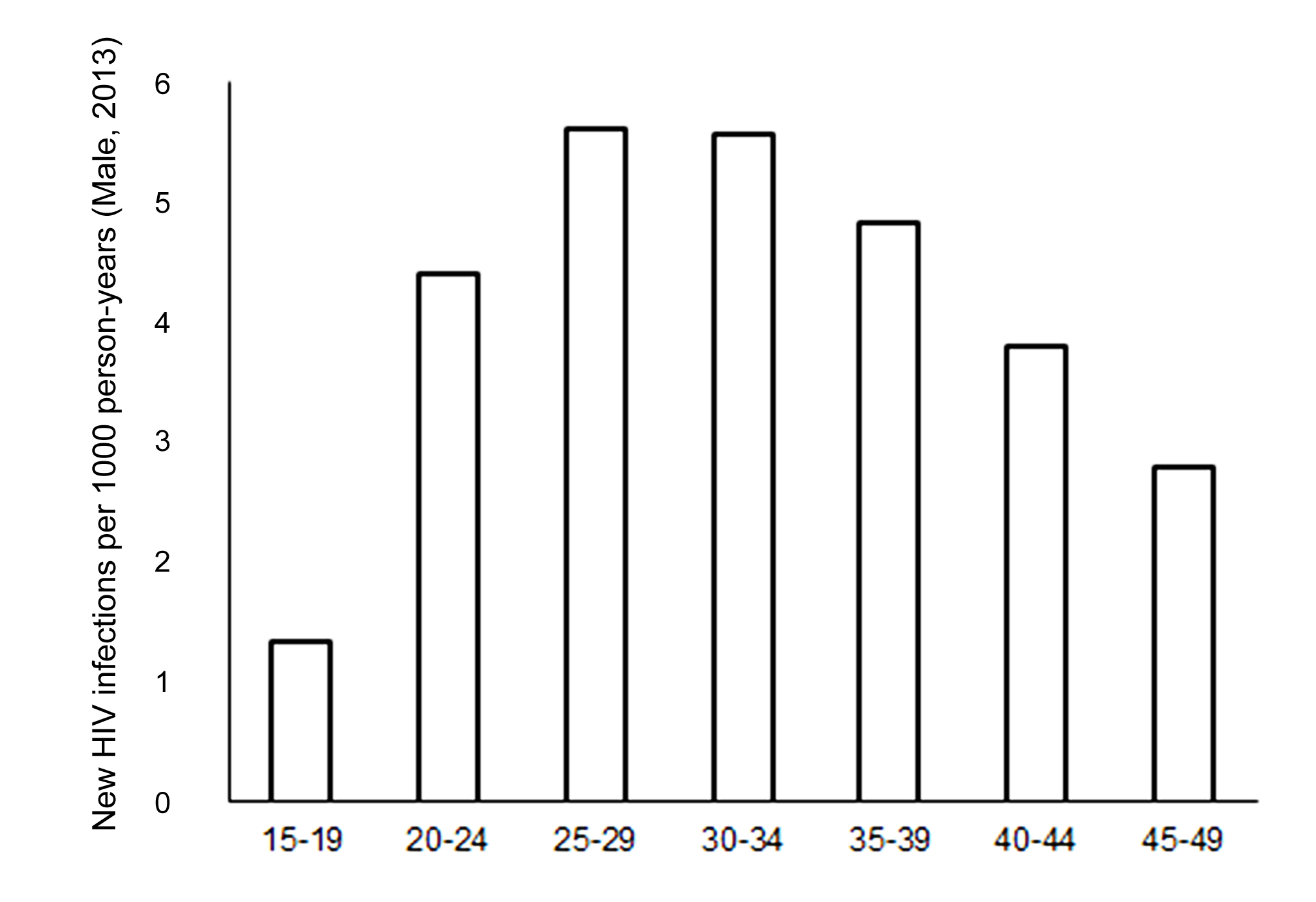

Supplement: S1 Fig — (TIF) [file pone.0153363.s003.tif]

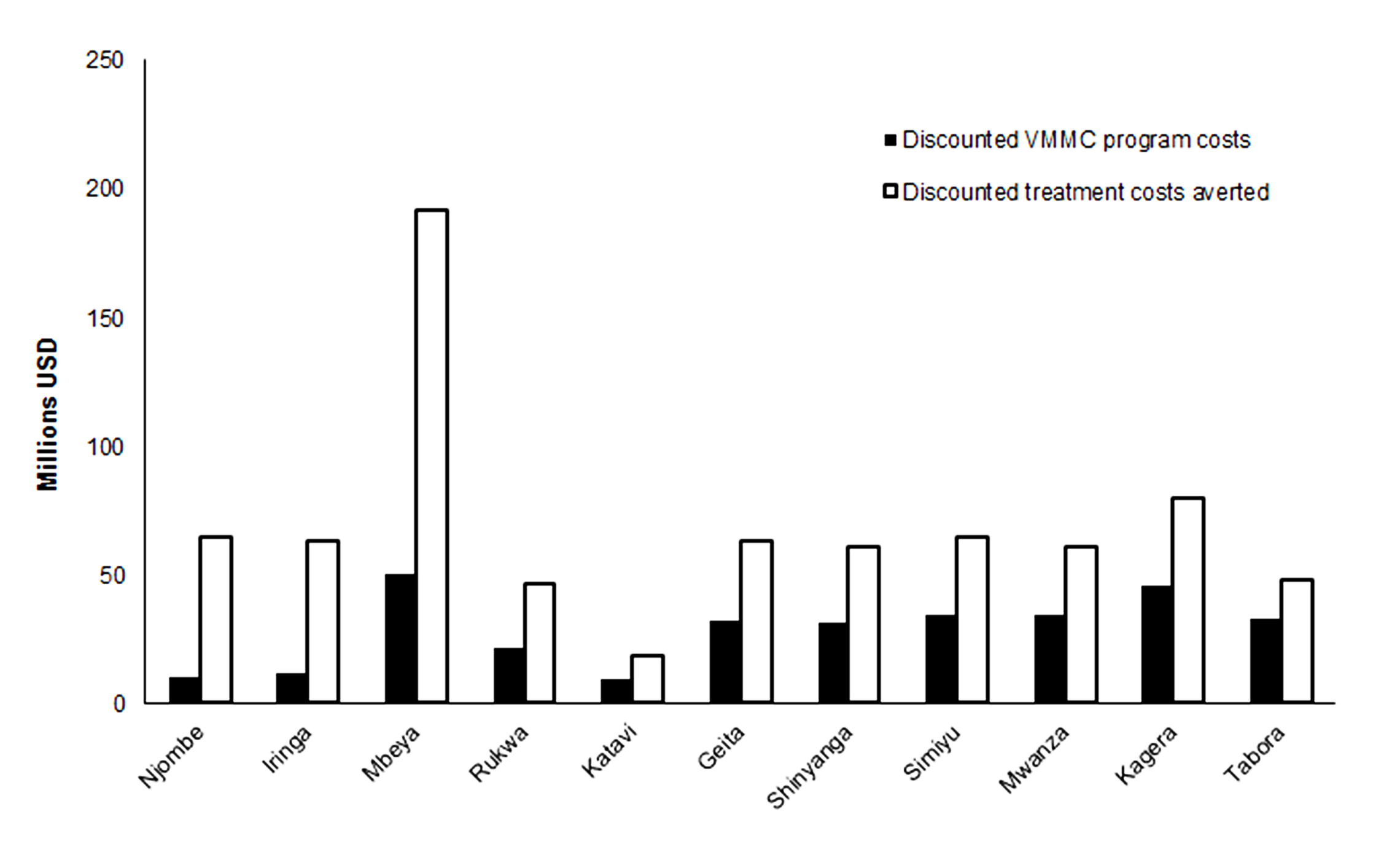

Supplement: S2 Fig — (TIF) [file pone.0153363.s004.tif]
